# *PRRX1* Is a Novel Prognostic Biomarker and Facilitates Tumor Progression Through Epithelial–Mesenchymal Transition in Uveal Melanoma

**DOI:** 10.3389/fimmu.2022.754645

**Published:** 2022-02-25

**Authors:** Zhishang Meng, Yanzhu Chen, Wenyi Wu, Bin Yan, Lusi Zhang, Huihui Chen, Yongan Meng, Youling Liang, Xiaoxi Yao, Jing Luo

**Affiliations:** ^1^ Department of Ophthalmology, The Second Xiangya Hospital, Central South University, Changsha, China; ^2^ Department of Radiation Oncology, Hunan Cancer Hospital and The Affiliated Cancer Hospital of Xiangya School of Medicine, Central South University, Changsha, China; ^3^ Department of Ophthalmology, Xiangya Hospital, Central South University, Changsha, China; ^4^ Shenzhen College of International Education, Shenzhen, China

**Keywords:** uveal melanoma, *PRRX1*, tumor microenvironment, epithelial–mesenchymal transition, biomarker

## Abstract

Uveal melanoma (UM) is the most common primary intraocular malignancy in adults. UM develops and is sustained by inflammation and immunosuppression from the tumor microenvironment (TME). This study sought to identify a reliable TME-related biomarker that could provide survival prediction and new insight into therapy for UM patients. Based on clinical characteristics and the RNA-seq transcriptome data of 80 samples from The Cancer Genome Atlas (TCGA) database, *PRRX1* as a TME- and prognosis-related gene was identified using the ESTIMATE algorithm and the LASSO–Cox regression model. A prognostic model based on *PRRX1* was constructed and validated with a Gene Expression Omnibus (GEO) dataset of 63 samples. High *PRRX1* expression was associated with poorer overall survival (OS) and metastasis-free survival (MFS) in UM patients. Comprehensive results of the prognostic analysis showed that *PRRX1* was an independent and reliable predictor of UM. Then the results of immunological characteristics demonstrated that higher expression of *PRRX1* was accompanied by higher expression of immune checkpoint genes, lower tumor mutation burden (TMB), and greater tumor cell infiltration into the TME. Gene set enrichment analysis (GSEA) showed that high *PRRX1* expression correlated with angiogenesis, epithelial–mesenchymal transition (EMT), and inflammation. Furthermore, downregulation of *PRRX1* weakened the process of EMT, reduced cell invasion and migration of human UM cell line MuM-2B *in vitro*. Taken together, these findings indicated that increased *PRRX1* expression is independently a prognostic factor of poorer OS and MFS in patients with UM, and that *PRRX1* promotes malignant progression of UM by facilitating EMT, suggesting that *PRRX1* may be a potential target for UM therapy.

## Introduction

Uveal melanoma (UM) is the most common primary intraocular malignancy in adults, with no effective treatments currently available for metastatic UM (1). UM arises from the uveal tract of the eye, which contains the ciliary body, choroid, and iris. The clinical and biologic features of UM differ from cutaneous melanoma (2). The central mechanisms of UM progression and metastasis include aberrant gene expression, chromosomal abnormalities, cytokine imbalance, and dysregulation of signaling pathway (3–6). Primary UM can be treated with either radiation or surgery and has a low local recurrence rate. However, up to 50% of UM are aggressive malignancies, giving rise to liver metastases, with liver metastasis being a frequent cause of morbidity and mortality in patients with UM. The average overall survival (OS) time of untreated patients has been reported to be approximately 2 months (7, 8), whereas the median OS time with aggressive treatment ranged from 6 to 12 months (8–10).

UM develops from and is sustained by inflammation and immunosuppression in the tumor microenvironment (TME) (11, 12). In contrast to other solid tumors, increasing evidence indicates that high levels of tumor-infiltrating lymphocytes (TILs) in UM correlate with poor prognosis (13, 14). Vascularization, an essential feature of metastasis, is greater in UMs with moderate or intense immune infiltrates than those with a lower degree of immune infiltration (15). In addition, the stroma facilitates the proliferation and survival of tumor cells and promotes epithelial–mesenchymal transition (EMT), EMT of tumor cells can be induced by stimuli from the TME, resulting in metastasis and death (16, 17). Several EMT-inducing transcription factors (EMT-TFs) are identified to play central roles in the execution of EMT, even in non-epithelial tumors (melanoma, glioblastoma and leukemia) (18). Furthermore, the eye has been regarded as an immune-privileged area and UM has one of the lowest mutation burdens and leukocyte fractions among adult tumors, specific immune phenotypes that can rationally explain the poor efficiency of immune responses in these patients (2, 15). Current treatments of UM include surgery and radiotherapy (19, 20), although various immunotherapeutic agents have undergone clinical trials in patients with UM (8, 9, 21). UM is genetically and clinically distinct from cutaneous melanoma, it is largely unresponsive to immune checkpoint inhibitors (ICIs) (7, 22), with no effective immunotherapy or targeted therapy currently available for the treatment of UM (23).

The present study sought to identify a reliable biomarker for UM that can predict patient prognosis and responses to treatment. Transcriptome data were investigated and potential key genes of UM screened in the TCGA database. TME- and prognosis-related genes were evaluated using bioinformatics tools and algorithms. Further validation in another independent dataset and analysis of immunologic characteristics, as well as *in vitro* studies indicated that *PRRX1* is a novel prognostic biomarker and a promising therapeutic target in UM.

## Materials and Methods

### Patients and Datasets

Transcriptome RNA-sequencing and clinical data were from the TCGA-UVM dataset (https://portal.gdc.cancer.gov/) (24). Additionally, gene expression and clinical information files of GSE22138 were downloaded from the Gene Expression Omnibus (GEO) database (https://www.ncbi.nlm.nih.gov/geo/) (5).

### Identification of TME and Prognosis Related Hub Genes

Based on the RNA-seq data of 80 samples from the TCGA-UVM dataset, the “ESTIMATE” R package v1.0.13 was used to calculate ESTIMATE, immune, and stromal scores in the TME for all samples (25). The samples were divided into two groups based on the medians of these three scores. Thereafter, Kaplan–Meier analysis and the log-rank test (significance level: *p* < 0.05) were used to compare overall survival (OS) in two groups of patients using the R packages “survminer” v0.4.9 and “survival” v3.2-13.

Afterward, the “limma” package v3.48.3 in R was used to assess differential gene expression in the high and low immune/stromal score groups. The criteria for selecting differentially expressed genes (DEGs) included |log2(fold change)| >1 and a false discovery rate (FDR)-adjusted *p <*0.05. Results from these analyses were plotted as volcano plots using the “ggplot2” package v3.3.5 in R. Co-upregulated and co-downregulated DEGs in the high and low immune/stromal score groups were regarded as TME- and prognosis-related genes. These genes were subjected to the Gene Ontology (GO) functional enrichment and the Kyoto Encyclopedia of Genes and Genomes (KEGG) pathway analysis, with the results visualized using the R packages “clusterProfiler” v4.0.5 and “enrichplot” v1.12.2 (*p <*0.05, *q <*0.05).

Univariate Cox regression analyses (significance level: *p *<0.05) were performed to assess the relationships between the levels of expression of these potential candidate genes and OS in patients with UM, followed by least absolute shrinkage and selection operator (LASSO) regression analysis using the “glmnet” package v4.1-2 in R to avoid overfitting. This was followed by multivariate Cox regression analysis (significance level: *p *<0.05) to determine whether there was any independent prognostic effect of these candidate genes on OS.

### Survival Analysis and Assessment of *PRRX1* for Prognostic Prediction

Patients in the TCGA and GEO cohorts were divided into those with high and low levels of expression of *PRRX1* based on the median levels of *PRRX1* in each dataset. OS in the TCGA cohort and metastasis-free survival (MFS) in the GEO cohort were analyzed by the Kaplan–Meier method and compared in each pair of groups by the log-rank test (significance level: *p *<0.05), using the “survival” package v3.2-13 in R. The predictive power of the model was evaluated using receiver operating characteristic (ROC) and Cox regression (significance level: *p *<0.05) analyses, as calculated using the R packages “survival” v3.2-13, “survminer” v0.4.9 and “timeROC” v0.4. The prognostic impact of *PRRX1* expression in several types of cancer was investigated by pan-cancer analysis. RNA-seq data and clinical information come from patients with 33 types of tumors were downloaded from the TCGA database (https://portal.gdc.cancer.gov/). Univariate Cox regression (significance level: *p *<0.05) analysis was used to assess the relationship between OS and *PRRX1* expression, with the results displayed as forest plots using the “forestplot” package v2.0.1 in R. In addition, because cytogenetic studies have found that the loss of chromosome 3 (monosomy 3) and a gain of chromosome 8q are associated with UM metastasis (26) and the status of chromosomes 3 and 8q has been determined in every patient in the TCGA-UVM project (**Table S1**) (24), Spearman rank correlation analysis (significance level: *p *<0.05) was performed to assess the correlation between the number of copies of these chromosomes and *PRRX1* expression.

### Enrichment Analysis of *PRRX1* in UM

To determine the biological states and processes that correlate with *PRRX1* expression in UM, RNA-seq data from the TCGA-UVM dataset were analyzed by gene set enrichment analysis (GSEA) v4.1.0 software using the Hallmark gene sets (http://www.broadinstitute.org/gsea), with significance set at FDR <0.25 and *p <*0.05.

### Correlation Between *PRRX1* and Immunological Characteristics

The level of expression of immune checkpoint genes may correlate with responses to treatment with immune checkpoint inhibitor therapies (27). The immune checkpoint genes expressed in UM include *CTLA4*, *PD1* (*PDCD1*), *PDL1* (*CD274*), *TIGIT*, and *LAG3* (2, 11, 24). Therefore, the correlation between *PRRX1* expression and the expression of these critical immune checkpoint molecules was subsequently explored using the “ggplot2” v3.3.5 and “reshape2” v1.4.4 packages in R. In addition, the correlation between *PRRX1* expression and tumor mutation burden (TMB) was analyzed by Spearman’s correlation analysis (significance level: *p *<0.05). To quantify the composition of TME in UM, the abundance of TME-related cell populations was estimated from gene expression data using the “MCPcounter” package v1.2.0 in R.

### Cell Line

The human UM cell line MuM-2B purchased from iCell Bioscience Inc. (iCELL-h148;Shanghai, China) was chosen for its highly aggressive phenotype (28, 29). Cells were cultured in RPMI-1640 medium with 10% FBS and 1% penicillin–streptomycin (Gibco, USA) at 37°C with 5% CO_2_ condition. Experiments were performed with cells in the logarithmic growth period.

### siRNA Knockdown of *PRRX1 In Vitro*

*PRRX1* small interfering (si) RNA (si-*PRRX1*) and negative control (si-NC) oligonucleotide were purchased from RiboBio (Ribobio, Guangzhou, China) with the sequences GGAATAGGACAACCTTCAA (Si1), ACACTATCCTGATGCTTTT (Si2), and GTTCCGCAGGAATGAGAGA (Si3). Transient transfection with siRNA at a final concentration of 100 nM was performed with Lipofectamine 2000 (Invitrogen, USA) according to manufacturer’s protocol.

### Western Blotting

Total protein from cells was harvested using radio immunoprecipitation assay buffer (RIPA; Beyotime, China), and was quantified by BCA Assay (Beyotime, China). In brief, protein samples were separated by SDS–polyacrylamide gel electrophoresis and transferred onto polyvinylidene fluoride (PVDF) membranes, which was then incubated with primary antibodies and HRP-conjugated secondary antibodies (**Table S2**). Band intensity was quantified by Quantity One software (Bio-Rad) and defined as the ratio of target protein to β-actin. The assay was repeated three times.

### Cell Migration and Invasion Assays

The migration of UM cells was evaluated with a wound healing scratch assay. Cells were seeded in 6-well plates (5 × 10^5^/well) until reached full confluence. Cells were serum-starved overnight and an artificial scratch wound was created at the center of the well and photographed, the scratch areas were photographed at 0, 24 and 48 h. Migration index was calculated as the follows: [(original scratch width-scratch width at 24/48 h)/(original scratch width)] × 100%. The assay was repeated three times.

Transwell invasion assays were performed to assess cell invasion. 24-well Millicell hanging cell culture inserts (8.0 µm, Millipore, USA) and Matrigel (BD Biosciences, USA) were used as per manufacturer’s protocol. Approximately 2 × 10^5^ cells in serum-free medium (100 ul, 2 × 10^6^ cells/ml) were added per well to the upper chamber, 500 ul complete medium with 10% FBS in the lower chamber served as a chemoattractant. After 48 h of incubation, cells that had invaded through the matrigel were fixed in 4% paraformaldehyde and stained with 0.1% crystal violet for counting. Three separate fields were counted for each filter with microscope (UOP-DSZ2000X; Chongqing, China). The assay was also repeated three times.

## Results

### Process of Analyzing Patients and Datasets

**Figure 1:**
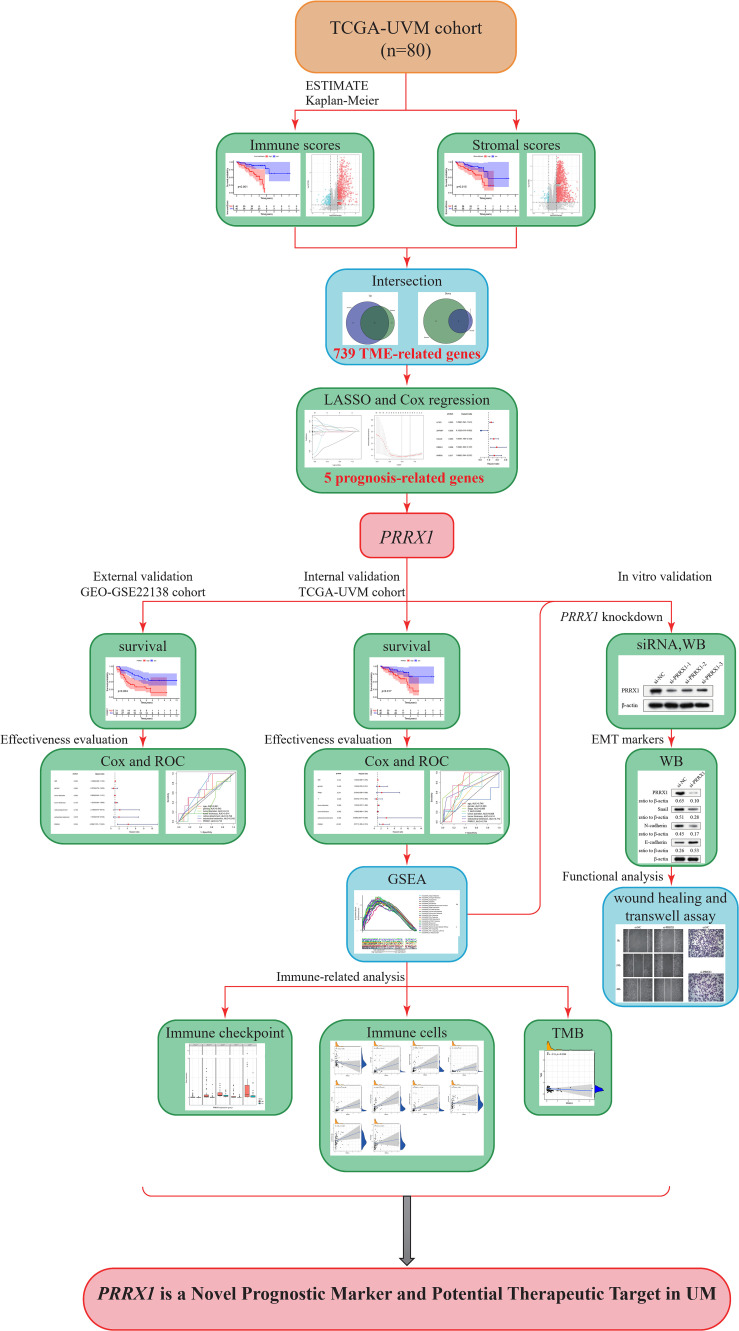
Study flow diagram. UVM/UM, uveal melanoma; TCGA, The Cancer Genome Atlas; TME, tumor microenvironment; LASSO, least absolute shrinkage and selection operator; PRRX1, paired related homoeobox 1; ROC, receiver operating characteristic; WB, Western blot; siRNA, small interfering RNA; GSEA, Gene Set Enrichment Analysis; GEO, Gene Expression Omnibus; TMB, tumor mutation burden.

A brief flowchart of our study is shown in **Figure 1**. Clinical data and transcriptome expression profiles of 80 patients were downloaded from the TCGA-UVM cohort. Similarly, information about the 63 patients in the validation cohort (GEO-GSE22138) was obtained from the GEO database. The clinical characteristics of these 143 patients are displayed in **Table 1**.

**Table 1:** Baseline characteristics of UM patients included in this study.

	TCGA-UVM cohort (n = 80) Number (%)	GEO-GSE22138 Cohort (n = 63) Number (%)
**Age (years)**		
≤60	40 (50.00)	28 (44.44)
>60	40 (50.00)	35 (55.56)
**Gender**		
Female	35 (43.75)	24 (38.10)
Male	45 (56.25)	39 (61.90)
**Stage**		
I	0 (0.00)	NA
II	36 (45.00)	NA
III	40 (50.00)	NA
IV	4 (5.00)	NA
**T classification**		
T1	0 (0.00)	NA
T2	4 (5.00)	NA
T3	36 (45.00)	NA
T4	38 (47.50)	NA
Unknown	2 (2.50)	NA
**N classification**		
N0	76 (95.00)	NA
N1	0 (0.00)	NA
Unknown	4 (5.00)	NA
**M classification**		
M0	73 (91.25)	28 (44.44)
M1	3 (3.75)	35 (55.56)
Unknown	4 (5.00)	0 (0.00)
**Tumor diameter (mm)**		
≤18	53 (66.25)	40 (63.49)
>18	26 (32.50)	13 (20.64)
Unknown	1 (1.25)	10 (15.87)
**Tumor thickness (mm)**		
≤10	37 (46.25)	12 (19.05)
>10	43 (53.75)	51 (80.95)
**Radiation therapy**		
Yes	3 (3.75)	NA
No	63 (78.75)	NA
Unknown	14 (17.50)	NA
**Extrascleral extension**		
Yes	7 (8.75)	5 (7.94)
No	68 (85.00)	48 (76.19)
Unknown	5 (6.25)	10 (15.87)
**Retinal detachment**		
Yes	NA	36 (57.14)
No	NA	22 (34.92)
Unknown	NA	5 (7.94)

NA, Not applicable.

### TME- and Prognosis-Related Hub Genes

**Figure 2:**
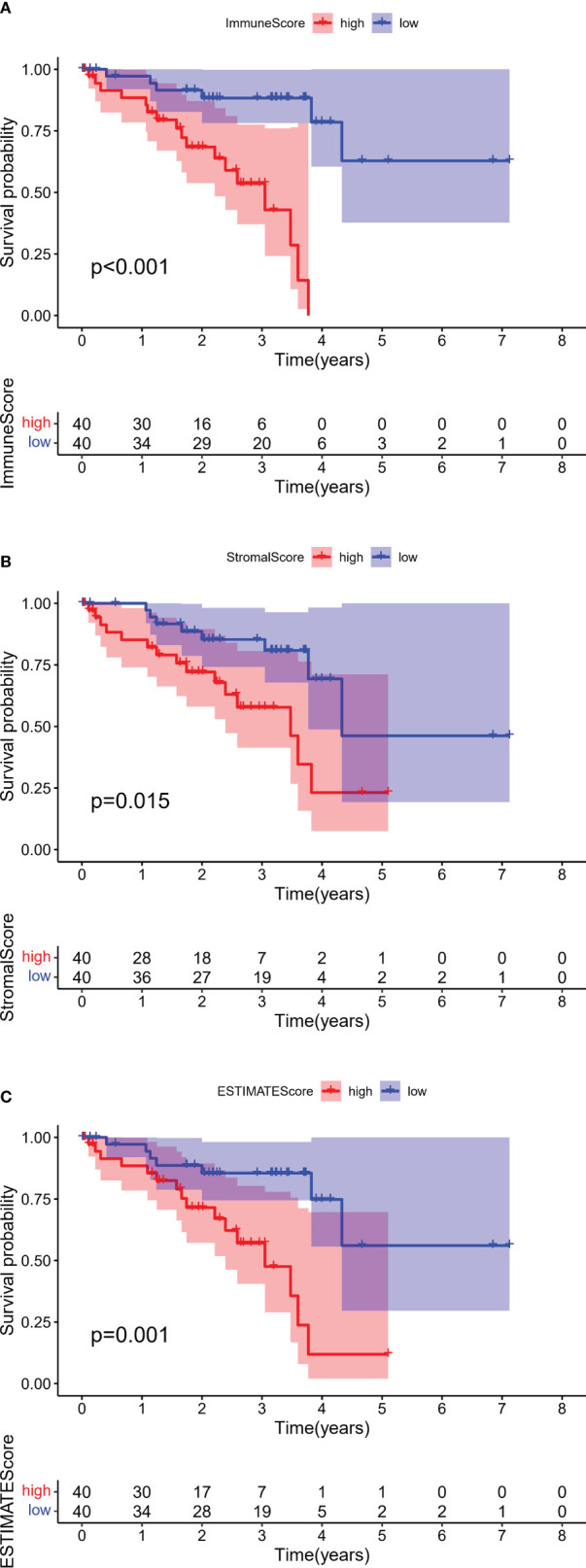
Kaplan–Meier survival analyses of overall survival in UM with high (red line) and low (blue line). **(A)** Immune scores, **(B)** Stromal scores, and **(C)** ESTIMATE scores. The numbers shown below the survival curves are the number of patients at risk at the specified year. The shaded red or blue represent 95% confidence intervals.

After assessment using an algorithm, the immune scores, stromal scores, and ESTIMATE scores for 80 samples in the TCGA-UVM cohort were calculated. For each scoring system, 40 samples each were classified into the high and low score groups based on the median scores, and OS was compared in each pair of samples. OS was found to be significantly lower in patients with higher immune scores (*p <*0.001, log-rank test; **Figure 2A**), stromal score (*p* = 0.015, log-rank test; **Figure 2B**) and ESTIMATE scores (*p* = 0.001, log-rank test; **Figure 2C**), indicating that immune and stromal components in the TME are associated with poor prognosis in patients with UM.

**Figure 3:**
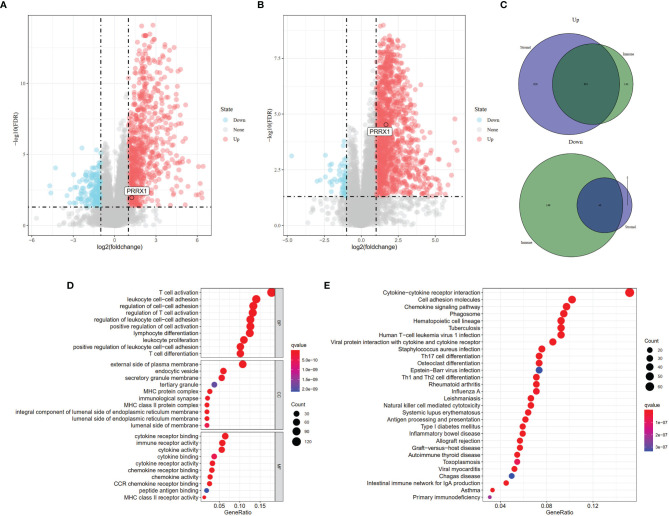
Identification of DEGs based on different scoring systems and related functional annotations. Analysis of DEGs based on **(A)** Immune scores (FDR <0.05, |log2(fold change)| >1 and **(B)** Stromal scores (FDR <0.05, |log2(fold change)| >1). Red or blue dots represent genes that were significantly upregulated or downregulated in each comparison (high *vs.* low). **(C)** Identification of the 739 overlapping genes commonly upregulated or downregulated in the two scoring systems. **(D)** GO enrichment analysis (*p <*0.05, *q <*0.05) and **(E)** KEGG pathway enrichment analysis (*p <*0.05, *q <*0.05) of the 739 overlapping genes. Dots size represented the number of enriched genes, the color represented *q*-value; the redder the color, the smaller the *q*-value.

To identify genes that might be responsible for these differences in OS, gene expression data were analyzed in 80 patients from the TCGA-UVM dataset. These analyses identified 1,031 DEGs (834 upregulated and 197 downregulated) between the high and low immune score groups (**Figure 3A**) and 1,367 DEGs (1,311 upregulated and 56 downregulated) between the high and low stromal score groups (**Figure 3B**). Comparison of these DEGs found that 739 overlapped, comprised of 48 co-down regulated genes and 691 co-upregulated genes (**Figure 3C**). These findings suggested that these 739 genes were likely to be key factors that affect both the prognosis and TME of UM. Furthermore, GO enrichment analysis of these 739 genes found that several GO terms were enriched in immune activity. The ten most significant terms in biological processes (BP), molecular functions (MF), and cellular components (CC) are shown in **Figure 3D**. KEGG analysis also showed that these DEGs mainly involved immune-related pathways (**Figure 3E**), further indicating that the immune composition within the TME is significantly prognostic of survival in patients with UM.

**Figure 4:**
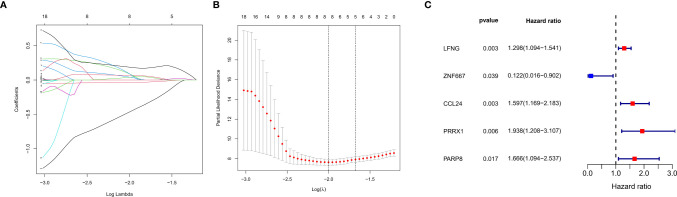
Construction of a prognostic gene signature using a LASSO–Cox regression model. **(A)** Trend graph of LASSO coefficients for 450 genes from univariate Cox regression analysis. the abscissa represents the log value of the optimal parameter (lambda), the ordinate represents the coefficient of the variable. **(B)** Attainment of lambda by a minimum combination of eight genes. Dotted vertical lines were drawn at the optimal values by using the minimum criteria and the 1 standard error (SE) of the minimum criteria (the 1−SE criteria). **(C)** Multivariate Cox analysis of previously screened genes, red dots represent the possible risk factors while blue represents potential protective factors.

Univariate Cox regression analysis showed 450 genes potentially prognostic of survival in patients with UM (**Table S3**). These genes were input into the LASSO Cox regression model (**Figure 4A**), with optimal performance attained by a combination of eight genes (*CTF1*, *LFNG*, *ZNF667*, *ARC*, *CCL24*, *PRRX1*, *PARP8*, and *ISG20*) (**Figure 4B**). Multivariate Cox analysis further showed that five of these genes (*LFNG*, *ZNF667*, *CCL24*, *PRRX1*, and *PARP8*) were independently prognostic of survival in patients with UM (**Figure 4C**). Previous studies have demonstrated that *PRRX1*, serve as a EMT-TF, is involved in the EMT process and tumor progression in several cancers (30–32), however, the role of *PRRX1* in UM was not reported. We therefore chose to focus our analysis on *PRRX1*.

### Association Between *PRRX1* Expression and Unfavorable Prognosis in Patients With UM

**Figure 5:**
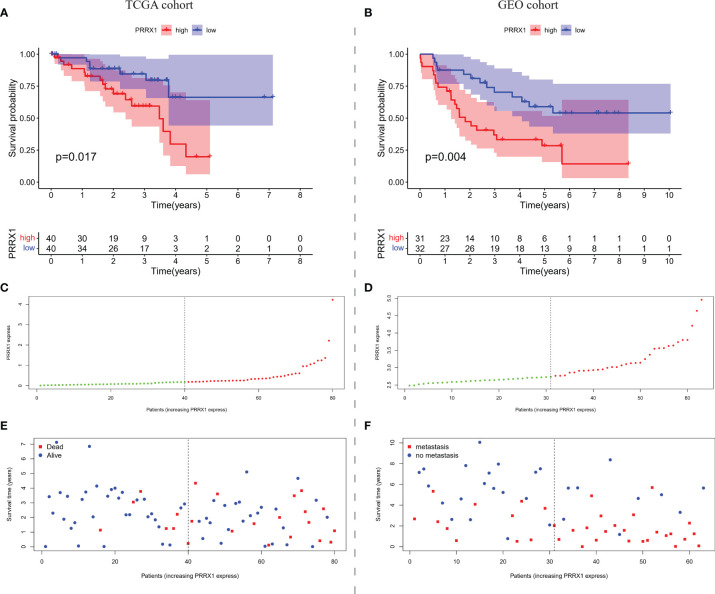
The overall prognostic performance of *PRRX1* expression in two UM cohorts. Kaplan–Meier analyses of **(A)** OS in TCGA cohort and **(B)** MFS in the GEO cohort, the numbers shown below the survival curves are the number of patients at risk at the specified year. Distribution of *PRRX1* expression levels in the **(C)** TCGA and **(D)** GEO cohorts, the dotted line represented the median *PRRX1* expression and divided the patients into low-expression (green dots) and high-expression groups (red dots). Percentages of **(E)** deceased patients (red dots) in the TCGA cohort and **(F)** patients with metastasis (red dots) in the GEO cohort as a function of *PRRX1* expression level.

Based on median *PRRX1* expression as the cut-off value, OS was found to be poorer in patients with high than low *PRRX1* expression (**Figure 5A**). The negative prognostic impact of higher *PRRX1* expression was validated in an independent GEO cohort, with MFS being significantly poorer with high than low *PRRX1* expression (**Figure 5B**). The distribution of *PRRX1* expression profiles is shown in **Figures 5C, D**. **Figures 5E, F** show the variations in patient survival and metastasis as a function of *PRRX1* expression. The percentages of deceased patients (**Figure 5E**) and patients with metastasis (**Figure 5F**) were greater and the survival time shorter in the high than in the low *PRRX1* expression group.

**Figure 6:**
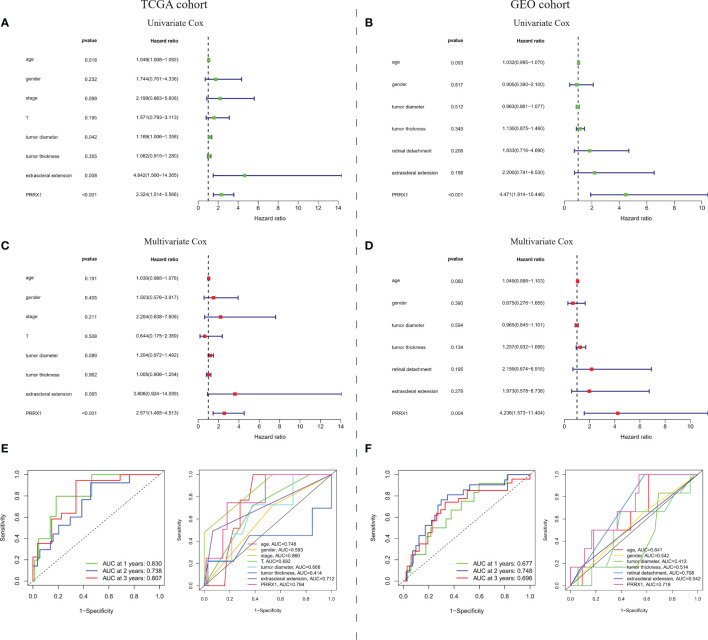
Prognostic predictive ability of *PRRX1* expression. **(A)** Univariate and multivariate Cox analyses of *PRRX1* expression and seven other clinicopathological parameters in the TCGA cohort. **(B)** Time-dependent ROC analysis for prediction of OS in the TCGA cohort. **(C)** ROC analyses of *PRRX1* expression and other seven clinicopathological parameters for predicting OS in the TCGA cohort. **(D)** Univariate and multivariate Cox analyses of *PRRX1* expression and six other clinicopathological parameters in the GEO cohort. **(E)** Time-dependent ROC analysis for prediction of MFS in the GEO cohort. **(F)** ROC analyses of *PRRX1* expression and other six clinicopathological parameters for predicting MFS in the GEO cohort. Corresponding AUC of each curve is displayed in the lower right corner of each figure, the higher the AUC value, the better the predictive power of the parameter.

To evaluate the prognostic predictive power of *PRRX1* expression, univariate and multivariate Cox analyses were performed to assess the ability of *PRRX1* expression to predict OS in the TCGA-UVM cohort and to predict MFS in the GEO-GSE22138 cohort. Both univariate (hazard ratio [HR]: 2.324, 95% confidence interval [CI]: 1.514–3.566, *p <*0.001) and multivariate (HR: 2.571 95% CI: 1.465–4.513, *p <*0.001) analyses of 75 patients in the TCGA-UVM cohort with complete information on age, gender, stage, T classification, tumor diameter, tumor thickness, and extra scleral extension showed that high *PRRX1* expression was a powerful and independent prognostic predictor of reduced OS (**Figure 6A**). The areas under the time-dependent ROC curves for 1-, 2-, and 3-year OS were 0.830, 0.738, and 0.870, respectively (**Figure 6B**). The area under the ROC curve (AUC) of *PRRX1* expression for predicting OS was 0.794 (**Figure 6C**), which was less than that for the stage but ranked second among evaluation metrics. Similarly, both univariate and multivariate analyses of the 42 patients in the GEO cohort with complete information, including, age, gender, tumor diameter, tumor thickness, retinal detachment, and extra scleral extension, showed that high expression of *PRRX1* was significantly predictive of reduced MFS (**Figure 6D**). The AUCs of the time-dependent ROC curves for 1-, 2-, and 3-year MFS were 0.677, 0.748, and 0.696, respectively (**Figure 6E**), and the AUC of *PRRX1* expression for predicting MFS was 0.718 (**Figure 6F**), greater than that for all other factors. These results indicate that *PRRX1* expression was an independent prognostic factor that could effectively predict the prognosis of UM patients.

**Figure 7:**
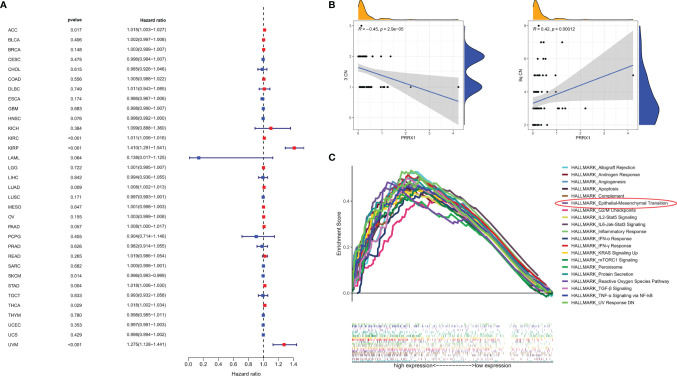
**(A)** Association between *PRRX1* expression level and OS in patients with 33 types of cancer from the TCGA database, red dots represent the possible risk factors while blue represents potential protective factors, abbreviations for all cancer types are in **Table S4**. **(B)** Correlations between *PRRX1* expression and chromosome 3/8q copy number, the density curve on the x and y-axes represents the distribution trend of themselves. The blue line indicates the best fitted linear models and the shaded area indicates the 95% confidence interval. **(C)** GSEA plot of the top 20 ranked Hallmark sets of genes associated with high *PRRX1* expression, as determined by comparing high and low *PRRX1* expression groups (FDR < 0.25, *p < *0.05).

Univariable Cox regression analysis was performed to assess the association of *PRRX1* expression levels with OS in patients with various types of cancer in 33 TCGA cohorts. These pan-cancer survival analyses indicated that *PRRX1* expression was an adverse prognostic factor in patients with seven types of cancer (**Figure 7A** **):** adrenocortical carcinoma (ACC), kidney renal clear cell carcinoma (KIRC), kidney renal papillary cell carcinoma (KIRP), lung adenocarcinoma (LUAD), stomach adenocarcinoma (STAD), thyroid carcinoma (THCA), and uveal melanoma (UVM). Assessment of the correlation between *PRRX1* expression and chromosome copy number using Spearman correlation analysis showed that *PRRX1* expression correlated negatively with chromosome 3 copy number but positively with chromosome 8q copy number (**Figure 7B**).

### GSEA of *PRRX1* in the TCGA-UVM Dataset

GSEA revealed that 26 Hallmark pathways were significantly enriched in patients with high *PRRX1* expression (**Table S5**). The top 20 ranked Hallmark pathways included the angiogenesis (*p* = 0.010, FDR = 0.005), epithelial–mesenchymal transition (*p <*0.001, FDR = 0.002), and TGF-β signaling (*p <*0.001, FDR = 0.002) pathways, and also various immune- and inflammation-related sets of genes, namely, inflammatory response, IFN-γ response, IL6-Jak-Stat3 signaling, IL2-Stat5 signaling, TNF-α signaling *via* NF-kB, and IFN-α response (**Figure 7C**). These results provide evidence for the possible phenotypes that may involve *PRRX1* expression.

### Immunological Characteristics of Different *PRRX1* Subgroups

**Figure 8:**
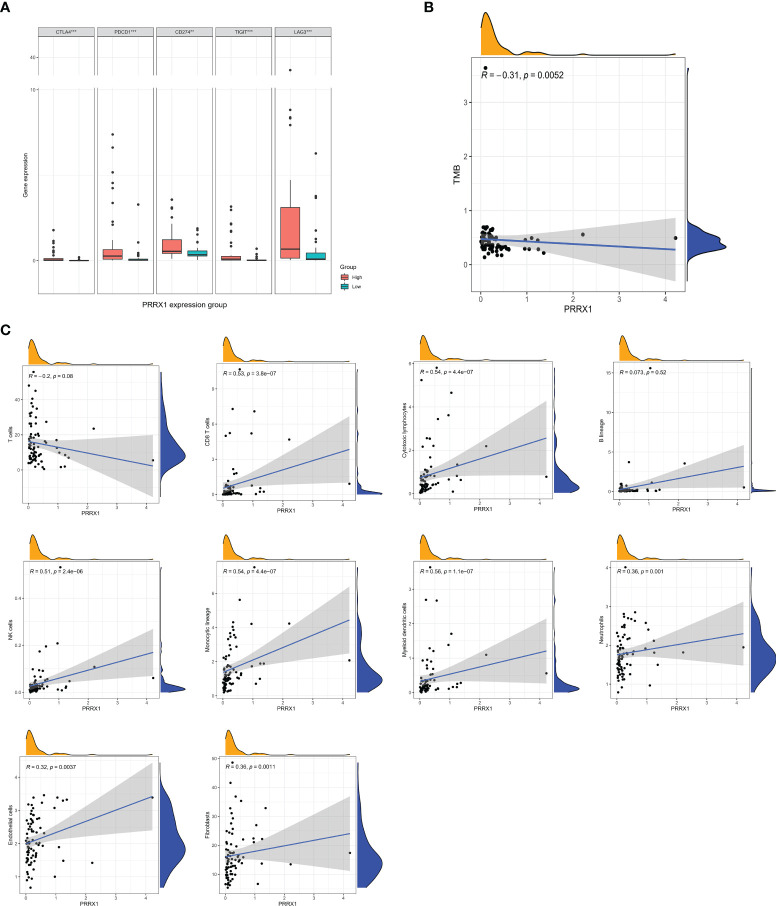
Analysis of immunologic characteristics associated with *PRRX1* expression levels. **(A)** Comparisons of the levels of expression of immune checkpoint genes in the high and low *PRRX1* groups (**p < 0.01; ***p < 0.001). **(B)** Correlation between *PRRX1* expression level and TMB. **(C)** Correlation between *PRRX1* expression and tumor immune/stromal cell populations in the UM microenvironment: T cells, CD8 T cells, cytotoxic lymphocytes, B lineage cells, NK cells, cells of the monocytic lineage, myeloid dendritic cells, neutrophils, endothelial cells, and fibroblasts, the x-axis represents the expression levels of *PRRX1*, and the y axis is the abundance of TME cells. The density curve on the x and y-axes represents the distribution trend of themselves. The blue line indicates the best fitted linear models and the shaded area indicates the 95% confidence interval.

The differences in expression of five immune checkpoint genes in the high and low *PRRX1* groups are shown in **Figure 8A**. The expression levels of all five genes in the high *PRRX1* expression group were significantly higher than that of the low expression group. TMB was found to decrease with increasing *PRRX1* expression (R = −0.31, *p* = 0.0052) (**Figure 8B**). Subsequent evaluation of the association between *PRRX1* expression and 10 TME-related cell populations from TCGA transcriptomic data using the Microenvironment Cell Populations-counter (MCPcounter) method (33, 34) showed that *PRRX1* expression was positively associated with eight types of cells: CD8 T cells, cytotoxic lymphocytes, NK cells, cells of the monocytic lineage, myeloid dendritic cells, neutrophils, endothelial cells, and fibroblasts (**Figure 8C**).

### Knockdown of *PRRX1* Inhibits UM Cell Invasion, Migration and EMT Progression

**Figure 9:**
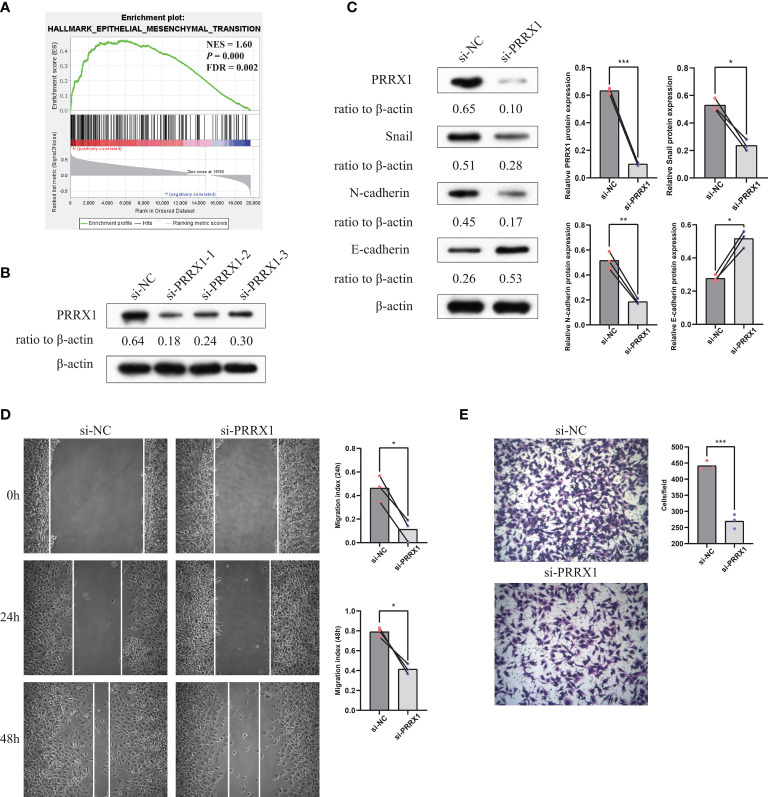
Knockdown of *PRRX1* in UM cell suppresses EMT, migration and invasion *in vitro*. **(A)** GSEA results revealed that *PRRX1* expression significantly positively correlated with the EMT signatures (NES = 1.6, *p < *0.001, FDR = 0.002). **(B)** WB showed PRRX1 protein level with three different *PRRX1*–siRNA treatment, relative expression levels of proteins were normalized based on the β-actin levels. **(C)** The influence of *PRRX1* knockdown on the expression level of EMT-related factors (Snail, N-cadherin and E-cadherin) were analyzed using WB, relative expression levels of proteins were normalized based on the β-actin levels (left: representative images of three independent experiments; right: quantitative analyses, n = 3, *p < 0.05; **p < 0.01; ***p < 0.001, paired Student’s t-test). **(D)** Wound-healing assay was used to detect cell migration ability in si-NC and si-*PRRX1* UM cells (left: representative images of three independent experiments; right: quantitative analyses, n = 3, *p < 0.05, paired Student’s t-test). **(E)** Transwell assays showed the effect of *PRRX1* knockdown on UM cell invasion (left: representative images of three independent experiments; right: quantitative analyses, n = 3, ***p < 0.001, unpaired Student’s t-test).

The previously stated GSEA results revealed that *PRRX1* expression significantly and positively correlated with the EMT signatures (**Figure 9A**). To investigate the biological function of *PRRX1* in UM progression, we then performed loss-of-function experiments that silence *PRRX1* in human UM cell line MuM-2B. The efficiency of the siRNA knockdown was confirmed by WB (**Figure 9B**), si-*PRRX1*-1 was chosen for subsequent experiments, which showed the most significant gene silencing efficiency. Compared to the control, downregulation of *PRRX1* upregulates E-cadherin expression and downregulates Snail and N-cadherin expression in UM cells (**Figure 9C**). Furthermore, results of transwell invasion and scratch assays also demonstrated that the knockdown of *PRRX1* significantly reduced UM cell migration and invasion (**Figures 9D, E**). Together, we initially confirmed *PRRX1* promotes UM progression through affecting the EMT process.

## Discussion

This study has provided new insights into *PRRX1* that could be a potential prognostic marker of survival in UM patients and therefore may be therapeutic targets. High immune scores, stromal scores, and ESTIMATE scores were associated with reduced OS, suggesting that a TME-related element was potentially able to evaluate prognosis in agreement with previous studies (30, 35, 36). GO and KEGG analyses suggest that the majority of TME and prognosis-related DEGs were involved in immune-related processes. *PRRX1* as a TME- and prognosis-related gene was identified using the ESTIMATE algorithm and the LASSO-Cox regression model. The subsequent focus on *PRRX1* showed that higher expression of this gene was associated with reduced OS and MFS in two independent datasets. Univariate and multivariate Cox analyses demonstrated that *PRRX1* was an independent biomarker of prognosis in UM, with ROC curve analysis showing that *PRRX1* can be classified as a robust predictor of UM. Furthermore, downregulation of *PRRX1* weakened the process of EMT, reduced cell invasion and migration of UM cells *in vitro*.

PRRX1 protein is a member of the family of paired-type homeobox transcription factors, which have important functions in the regulation of developmental morphogenetic processes, such as vasculogenesis and organogenesis (37). *PRRX1* is expressed in all known fibroblast subtypes. This protein modulates cutaneous fibrosis for the treatment of scarring and other pathologic fibrosis (38). Furthermore, *PRRX1* was shown to enhance the malignant properties of a wide variety of tumors. For example, *PRRX1*-mediated cancer-associated fibroblast (CAF) plasticity was found to have a significant impact on the biology and resistance to therapy of pancreatic ductal adenocarcinoma (31, 39). Overexpression of *PRRX1* was found to promote migration, invasion, and tumorigenesis and induce EMT in skin melanoma (40). To our knowledge, no previous study has assessed the relationships between *PRRX1* expression and UM. The present study showed that high *PRRX1* expression was associated with poor prognosis, high expression of immune checkpoint related gene and low TMB in UM patients. Clinical trials have reported that immune-based therapies are ineffective in the treatment of UM (41, 42). Our findings indicated that *PRRX1* is involved in the TME of UM, providing insight into the lack of efficacy of existing therapy for UM.

Several studies suggested the role of PRRX1 in the regulation of tumor progression and metastasis through the regulation of the EMT, and acting as a ETM-TF that regulates stemness activity and EMT plasticity in TME (17, 43). The *PRRX1* isoform *PRRX1A* was found to play important roles in regulating the metastatic potential and stemness of lung cancer (44), a finding supported by the results of our pan-cancer analysis. For instance, *PRRX1* isoforms were shown to be important in pancreatic ductal adenocarcinomas (PDAC), especially in the regulation of the EMT and the mesenchymal-to-epithelial transition (MET) in liver metastases of PDAC (31, 39). Although *PRRX1* was found to mediate carcinoma cell invasion and metastasis by inducing the EMT (37, 45, 46), other studies found that the loss of *PRRX1* results in the reversion of the EMT and the induction of the MET, favoring metastatic colonization (32, 47, 48). In this study, we found that knockdown of *PRRX1* reduces EMT-related proteins and inhibited UM cell migration and invasion. Further investigations of the deeper mechanisms by which *PRRX1* induces the EMT or MET in patients with primary and metastatic UM may provide a fuller understanding of the molecular mechanism associated with distant metastases, and also suggesting novel treatment strategies.

The present study also found that *PRRX1* expression was negatively correlated with chromosome 3 copy number, but positively correlated with chromosome 8q copy number. Several recent multi-omic studies have identified new molecular UM subsets, with types C and D being highly lethal. These subsets are characterized by the deletion of chromosome 3; the inactivation of *BRCA1*-associated protein 1 (*BAP1*), which is located on chromosome 3; and the gain of chromosome 8q (24, 49, 50). Our results support these findings, providing insights into the involvement of the *PRRX1* gene in the chromosomal abnormalities of UM. Type D UM is characterized by an inflammatory phenotype (51). Our *PRRX1*-related GSEA and TILs analyses showed that high expression of *PRRX1* was associated with inflammation, indicating that *PRRX1* affects the TME in patients with UM. Additional studies are needed to evaluate the relationships among aberrant expression of *PRRX1*, chromosomal abnormalities, and inflammation-related activities during UM progression and metastasis. Further characteristics of EMT process induced by *PRRX1* in the context of the changing TME of UM merit more attention in future studies.

An analysis of the relationship between *PRRX1* expression and 10 immune cell types in UM samples showed that the level of *PRRX1* expression was positively associated with invasion by eight immune cell types: CD8 T cells, cytotoxic lymphocytes, NK cells, cells of the monocytic lineage, myeloid dendritic cells, neutrophils, endothelial cells, and fibroblasts). These findings confirmed that *PRRX1* plays a significant role in the TME of UM. Previous studies have demonstrated that UM-infiltrating leukocytes mainly included macrophages and CD8^+^ T cells (13). T cell infiltrates with immunosuppressive activity have been associated with loss of *BAP1* and monosomy 3 (52, 53). We found that the levels of macrophages in UM tissue were positively correlated with *PRRX1* gene expression. M2-like macrophages, which constitute a large proportion of monocytes in UM (54, 55), are tightly associated with angiogenesis and vasculogenic mimicry (56, 57), suggesting that high *PRRX1* expression in UM was also associated with angiogenesis and vasculogenic mimicry. Moreover, *PRRX1* was shown to promote angiogenesis in gliomas by upregulating the expression of proangiogenic factors such as VEGF (58). Further investigation is required to assess the relationship between *PRRX1* expression and angiogenesis in patients with UM.

## Conclusion

In conclusion, this study showed that *PRRX1* expression affects UM progression and metastasis. *PRRX1* may therefore be a promising biomarker for UM that can predict patient prognosis and the efficacy of anticancer therapies. These findings may provide an individualized therapeutic approach for patients carrying the *PRRX1* variant.

## Data Availability Statement

The datasets analyzed during this study are available in TCGA database (https://portal.gdc.cancer.gov) and the Gene Expression Omnibus (GEO) database (https://www.ncbi.nlm.nih.gov/geo/).

## Author Contributions

Conceptualization: JL, ZM, and YC. Methodology and software: ZM, YC, and WW. Visualization: ZM, YC and XY. Formal analysis and data curation: BY, YM, YL, and XY. Writing: ZM and YC. Review and editing: WW, LZ, HC and JL. Supervision and acquisition of funding: JL. All authors listed have made a substantial, direct, and intellectual contribution to the work and approved it for publication.

## Funding

This work was supported by grants from the National Natural Science Foundation of China (81570847) and the Hunan Provincial Natural Science Foundation of China (2020JJ4800).

## Conflict of Interest

The authors declare that the research was conducted in the absence of any commercial or financial relationships that could be construed as a potential conflict of interest.

## Publisher’s Note

All claims expressed in this article are solely those of the authors and do not necessarily represent those of their affiliated organizations, or those of the publisher, the editors and the reviewers. Any product that may be evaluated in this article, or claim that may be made by its manufacturer, is not guaranteed or endorsed by the publisher.
