# Supplementary figures and images for: PRRX1 Is a Novel Prognostic Biomarker and Facilitates Tumor Progression Through Epithelial–Mesenchymal Transition in Uveal Melanoma
Source: Front Immunol. 2022 Feb 25;13:754645. doi: 10.3389/fimmu.2022.754645 (PMC8914230; doi:10.3389/fimmu.2022.754645)

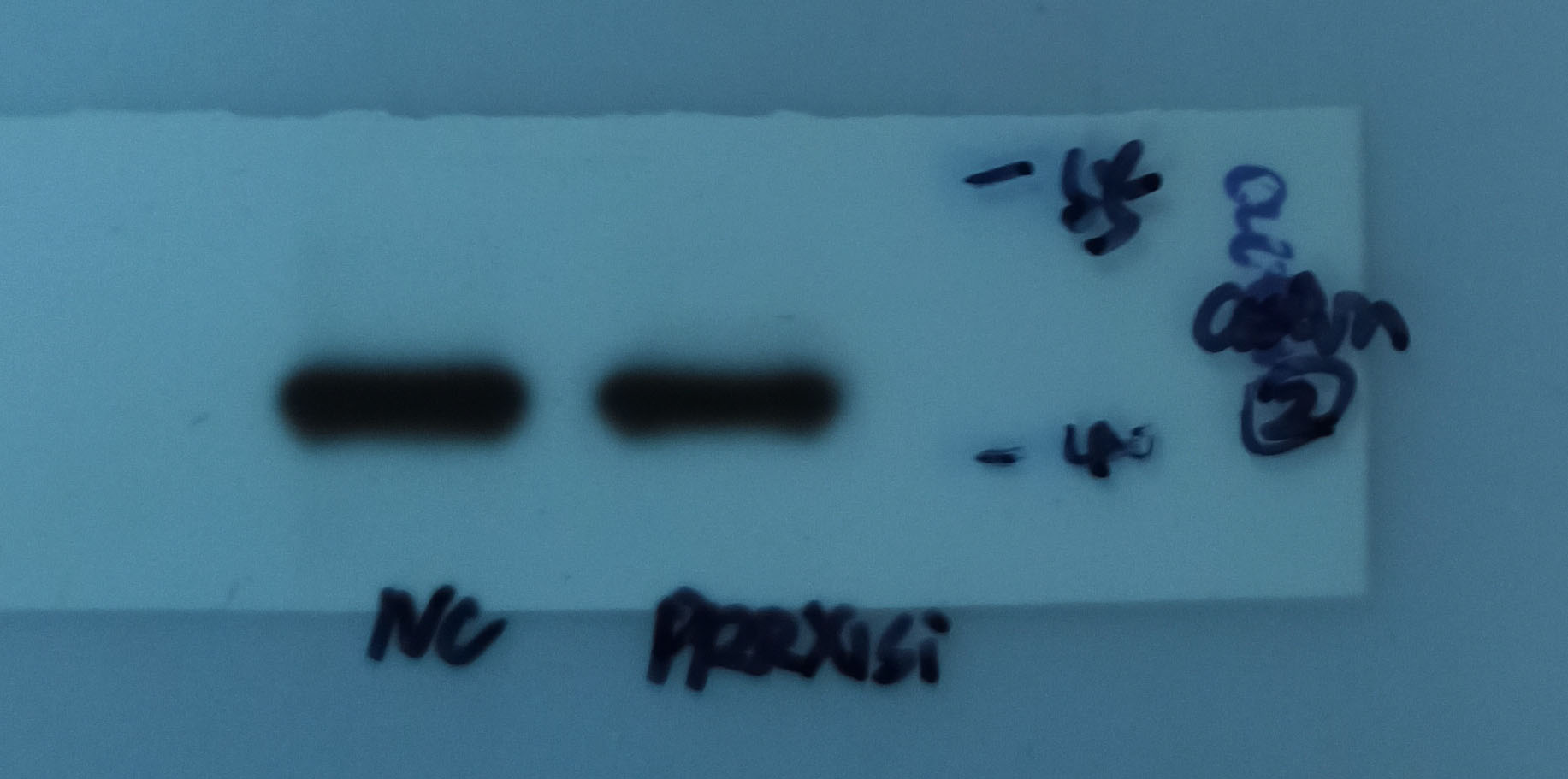

Supplement: Supplementary file 1 [file Image_1.tif]

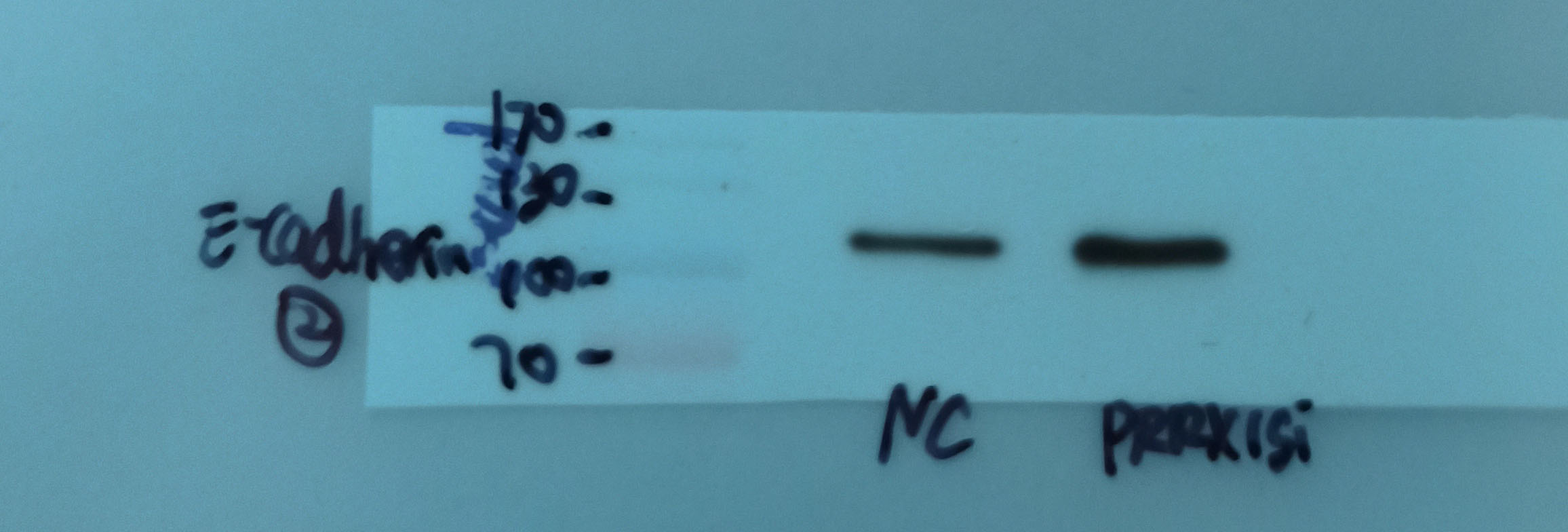

Supplement: Supplementary file 2 [file Image_2.jpeg]

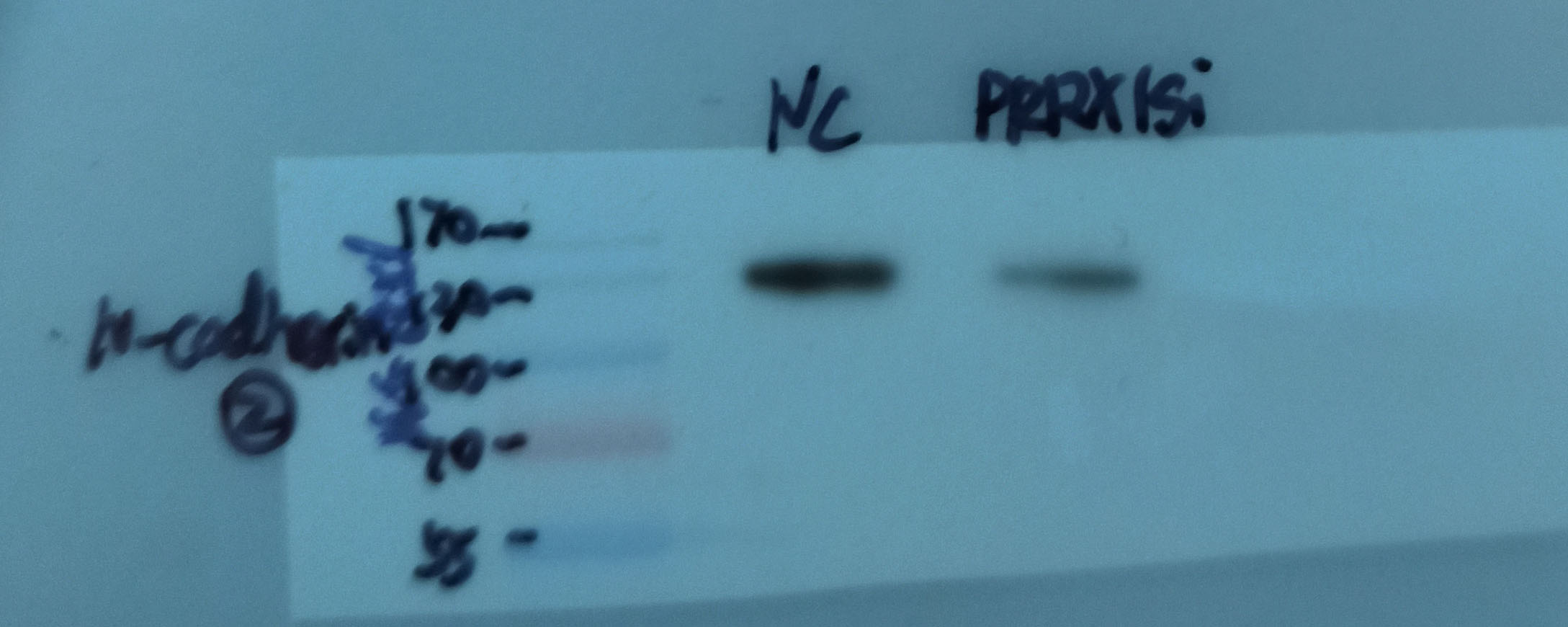

Supplement: Supplementary file 3 [file Image_3.jpeg]

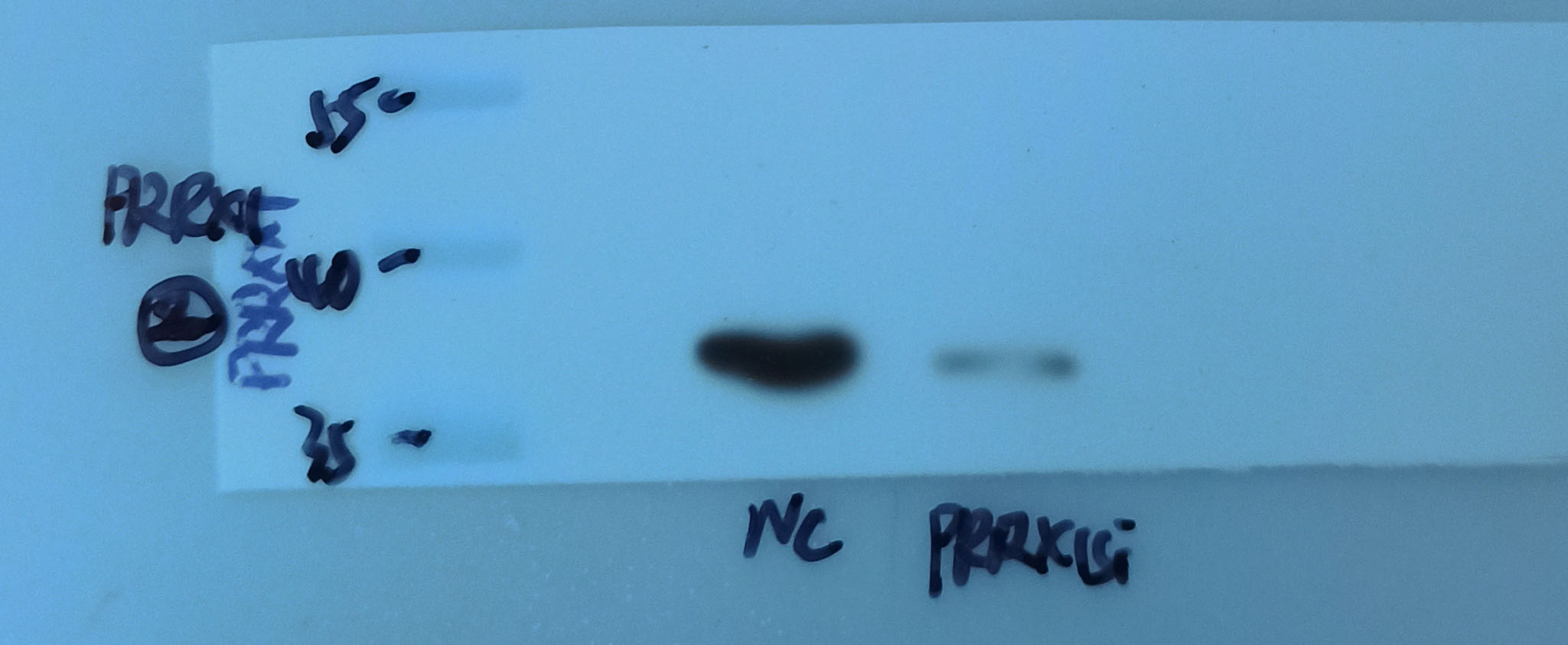

Supplement: Supplementary file 4 [file Image_4.jpeg]

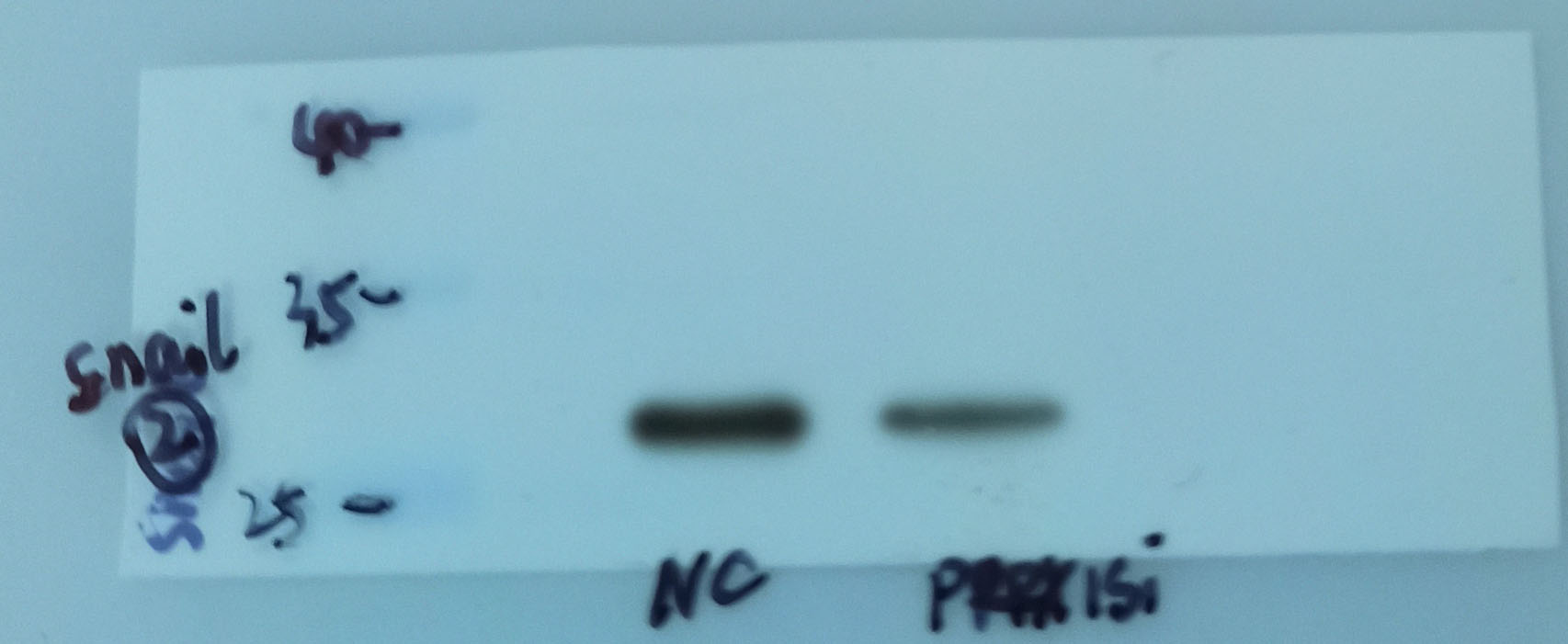

Supplement: Supplementary file 5 [file Image_5.jpeg]
