# Supplementary material for: PRRX1 Is a Novel Prognostic Biomarker and Facilitates Tumor Progression Through Epithelial–Mesenchymal Transition in Uveal Melanoma
Source: Front Immunol. 2022 Feb 25;13:754645. doi: 10.3389/fimmu.2022.754645 (PMC8914230; doi:10.3389/fimmu.2022.754645)
Supplement: Supplementary file 7 [file Table_2.docx]

| Names | Identifier | Dilution | Source |
| --- | --- | --- | --- |
| PRRX1 | TA803116S | 1：2000 | origene |
| E-cadherin | 20874-1-AP | 1：5000 | proteintech |
| N-cadherin | 22018-1-AP | 1：2000 | proteintech |
| Snail | #3879 | 1：1000 | CST |
| β-actin | 66009-1-Ig | 1：5000 | proteintech |
| HRP goat anti-mouse IgG | SA00001-1 |  | proteintech |
